# Personalised Treatment in Aortic Stenosis: A Patient-Tailored Transcatheter Aortic Valve Implantation Approach

**DOI:** 10.3390/jcdd9110407

**Published:** 2022-11-21

**Authors:** Andreas Mitsis, Xun Yuan, Christos Eftychiou, Panayiotis Avraamides, Christoph A. Nienaber

**Affiliations:** 1 Department of Cardiology, Nicosia General Hospital, Strovolos 2029, Cyprus; 2 Cardiology and Aortic Centre, Royal Brompton and Harefield Hospitals, Guy’s and St Thomas’ NHS Foundation Trust, London SW3 6NP, UK; 3 National Heart and Lung Institute, Faculty of Medicine, Imperial College London, London SW7 2BX, UK

**Keywords:** balloon expandable valves (BEV), self-expandable valves (SEV), surgical aortic valve replacement (SAVR), transcatheter aortic valve replacement (TAVR), transcatheter aortic valve implantation (TAVI)

## Abstract

Transcatheter aortic valve replacement (TAVI) has become a game changer in the management of severe aortic stenosis shifting the concept from inoperable or high-risk patients to intermediate or low surgical-risk individuals. Among devices available nowadays, there is no clear evidence that one device is better than the other or that one device is suitable for all patients. The selection of the optimal TAVI valve for every patient represents a challenging process for clinicians, given a large number of currently available devices. Consequently, understanding the advantages and disadvantages of each valve and personalising the valve selection based on patient-specific clinical and anatomical characteristics is paramount. This review article aims to both analyse the available devices in the presence of specific clinical and anatomic features and offer guidance to select the most suitable valve for a given patient.

## 1. Introduction

Transcatheter Aortic Valve Implantation (TAVI) has become a game changer in managing severe aortic stenosis. Over the last 20 years, many devices have emerged, but few have matured and dominated the market. The TAVI procedure has now been widely adopted, providing excellent short-term and long-term results, extending the concept from a treatment method for patients with prohibited surgical risk to the common practice even for patients with intermediate or relatively low surgical risk [1,2].

Among devices available nowadays, there is no clear evidence that one device is better than the other or that one device is suitable for all patients. Therefore, understanding the advantages and disadvantages of each valve and personalizing the valve selection based on patient-specific clinical and anatomical characteristics is paramount. This review article aims to both analyse the available devices and offer guidance to select the most suitable valve for a given patient.

## 2. Overview of the Available THVs

All the available transcatheter heart valves (THVs) belong to one of the following categories: balloon expandable (BEV) or self-expandable (SEV) valves. A BEV expands using the radial strength of the associated balloon. In contrast, a SEV deploys until it faces the resistance of the annular wall, adapting to the anatomy of the aortic annulus [3].

Another classification may follow the way the leaflets mount within the stented frame to the native aortic annulus. Based on this grouping, THVs can be classified as supra-annular and intra-annular valves. A supra-annular THV is designed to avoid intrusion with the native annulus preventing blood flow obstruction. In addition, a supra-annular THV leads to lower transvalvular gradients and higher effective orifice areas (EOA), allowing more extended durability [4]. On the other hand, an intra-annular THV is more similar to aortic anatomy and has the potential advantage of less interaction with coronary ostia, minimizing the risk of obstruction [5].

Overall, a SEV with supra-annular design is usually recommended in patients with small or severely calcific annulus, in cases of Valve in Valve (ViV) TAVIs, and in patients at risk for poor tolerance to rapid pacing, as ventricular pacing during implantation is not mandatory.

In the United States, the commercially available THVs (FDA approval) include the BEV SAPIEN 3™ and SAPIEN 3 Ultra™, and the SEV Evolut R™, Evolut PRO™, and PRO+™, as well the SEV Portico™ and Navitor™ valves. Of note, the FDA recently announced a recall of all unused inventory of the Boston Scientific LOTUS Edge™ Aortic Valve System due to complexities associated with the product delivery system. Approved valves in Europe (CE approval) include the preceding valves as well as, the SEV ACURATE Neo™ and Neo2™, and the BEV Myval™ valve.

### 2.1. SAPIEN Family BEV

**Figure 1:**
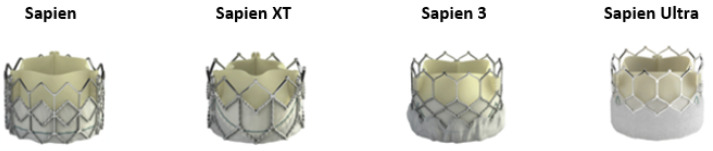
Evolution of the Sapien balloon-expandable valve. The device consists of a trileaflet, bovine pericardial tissue valve mounted on stainless-steel frame. The upper part has an open cell geometry to avoid obstruction and the lower part of the device is covered by a textured sealing polyethylene terephthalate material to reduce paravalvular leak.

The SAPIEN THV (Edwards Lifesciences Corporation, Irvine, CA, USA) was the family’s first generation THV, consisting of a trileaflet, bovine pericardial tissue valve mounted on stainless-steel frame (Figure 1). The Placement of Aortic Transcatheter Valves (PARTNER) A and B trials studied the SAPIEN THV and proved its efficacy in in-operable or high-risk (HR) patients [6,7]. The PARTNER 2A trial was a large, multicentre trial using the second-generation SAPIEN XT valve in intermediate-risk (IR) patients showing non-inferiority in death or disabling stroke at two years for patients that were treated with the SAPIEN-XT valve versus surgical aortic valve replacement (SAVR) [8].

The next generation of the Edwards BEV family was the SAPIEN 3 (S3) and was tested in the PARTNER 2-S3 trial. The study showed that the use of S3 was associated with a low early and 1-year complication rate and 1-year mortality in IR to HR inoperable patients treated with TAVI [9]. Finally, the SAPIEN 3 was examined in IR patients, showing that TAVI was similar to SAVR concerning the primary end point of death or disabling stroke [10].

The first major improvement of S3 was the introduction of a polyethylene terephthalate (PET) outer skirt designed to reduce paravalvular leak (PVL). The upper frame design with an open cell geometry was maintained to avoid obstruction and allow access to coronary arteries. The second significant development was an improved delivery system, the Edwards Commander Delivery System, compatible with the 14-French eSheath™ for the 20-mm, 23-mm, and 26-mm S3 valves and with the 16-French™ eSheath for the 29-mm S3 valve [9].

The latest generation of the SAPIEN BEV family is the SAPIEN-3 Ultra system. The S3 Ultra THV is based on the S3 valve platform but incorporates a 40% taller skirt designed to allow up to 50% more contact area with the native anatomy. In addition, the outer skirt was replaced by a textured sealing PET material, which was designed to promote enhanced healing and endothelialization. The new external skirt design has been introduced in 20-, 23-, and 26-mm SAPIEN 3 Ultra valves, while the 29-mm valve remains the current S3 model. The newest valve is compatible with the 14-French sheath for the 20, 23, and 26-mm sizes [11]. The S3 Ultra THV was approved by the U.S. Food and Drug Administration in late 2018 and obtained an expanded indication to treat low-risk patients in 2019. In addition, 30-day results of the S3 Ultra in a propensity score matching registry with the older S3 showed that device success rates were high with both BEVs, with overall low rates of adverse events up to 30 days after TAVI. With the S3 Ultra THV, despite the lower frequency of pre-dilatation, the major benefit was the significant reduction of mild PVL, and this confirmed the improved annular sealing properties of the novel S3 Ultra THV [12].

### 2.2. The CoreValve/Evolut Family SEV

**Figure 2:**
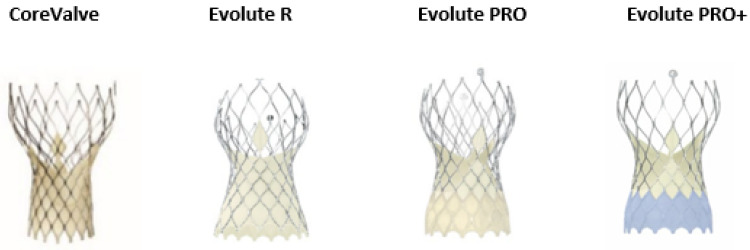
Evolution of the CoreValve/Evolute self-expandable valve. The device consists of self-expanding trileaflet porcine pericardial tissue on a nitinol frame with a larger cell size and a shorter frame height. The external tissue wrap provides advanced sealing.

The SEV supra-annular valve was initially presented by Medtronic’s CoreValve system (Medtronic Inc., Minneapolis, MN, USA), which consisted of self-expanding trileaflet porcine pericardial tissue on a Nitinol frame (Figure 2). The first study that compared the CoreValve vs. SAVR in high-risk patients was the CoreValve United States Pivotal Trial. This study found that TAVI was correlated with a considerably elevated survival rate at one year [13]. The main disadvantages of the first generation SEV were the relatively large size of the delivery system, which had increased the incidence of intraprocedural vascular complications, the need for permanent pacing post procedural, the increase rate of PVL, and the relatively elevated rates of strokes [14,15,16,17].

The Medtronic Evolut R valve followed this initial self-expandable THV. The new device managed to minimize the overall height by 10% of the prosthesis. In parallel, the new device maintained the height of the pericardial skirt and lengthened it to permit an increase seal against PVL. Additionally, the introduction of the novel built-in InLine sheath, with a reduced overall profile of the delivery system down to a 14-Fr equivalent sheath, allowed the insertion of the entire system without the demand of an additional access sheath. Many registries showed favourable long-term outcomes of the new device compared to the CoreValve system, mainly based on a significant reduction in PVL [18,19,20].

The next generation, the Evolute PRO, consisted of a porcine, pericardial, trileaflet valve sutured into a self-expanding Nitinol frame. This new THV kept all the features and the size of the Medtronic Evolut R, is recapturable and repositionable to assist in optimal deployment, but added an external porcine pericardial wrap over the first 1.5 cells, intending to reduce the incidence of PVL. In addition, the frame appeared to have larger cells and a shorter frame height alongside an approximately 10% reduction in length in the outflow segment. These changes allowed a better alignment in cases of horizontal aorta. There are 4 available sizes of the Evolut PRO valve. The 23-mm is suitable for aortic valve annuli between 18–20 mm, the 26-mm for annuli between 20–23 mm, the 29-mm for annuli between 23–26 mm and finally, the 34-mm is suitable to treat aortic valve annuli between 26–29 mm. The recommended implantation depth remains 3–5 mm below the aortic annulus, however nowadays many operators are aiming for an even higher implantation in order to minimize the risk of postprocedural permanent pacing [21,22].

The newest generation of the system is the Evolut PRO+ platform. The main improvement of this THV is the addition of an external part between the valve and the native aortic annulus to improve the valve sealing and reduce PVL. Additionally, the new version of the valve is one of the few commercially available TAVI system that can treat even larger annulus ranged up to 30 mm diameter. Finally, a last advantage is the miniaturization of the delivery system for access down to 5.0 mm vessels with 23–29 mm valves [23]. Of note, the latest member of the Evolute family, the Evolute FX valve has been recently developed, and designed to provide greater precision and control throughout the procedure, making the system easier to use with enhanced visualization capabilities for orientation and dept [24].

### 2.3. The ACURATE Neo/ACURATE Neo 2 SEV

**Figure 3:**
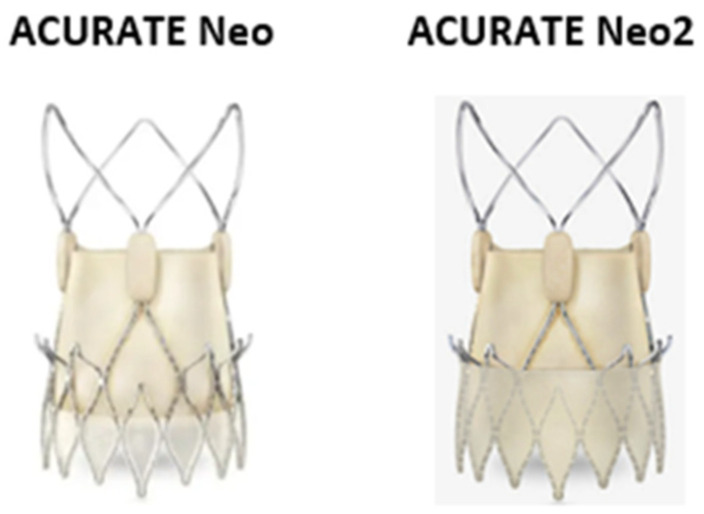
The ACURATE neo and neo2 self-expandable valve consist of a porcine pericardial tissue bio-prosthesis stitched into a self-expanding Nitinol stent and covered on the inside and outside with an anti-PVL porcine pericardial skirt. The open upper frame of the device open frame provides unrestricted access for future interventions.

The ACURATE Neo 2 (Boston Scientific, MA, USA), the evolution of the ACURATE Neo, is a SEV porcine pericardial tissue bio-prosthesis stitched into a self-expanding Nitinol stent (Figure 3). The valve is protected with an anti-PVL porcine pericardial skirt [25]. The valve has a supra-annular design and includes three stabilization arches for axial alignment, an upper crown for capping the aortic annulus, and a low crown that is opened over the native aortic valve for full deployment, following a top to bottom deployment. The ACURATE Neo2 is available in three different sizes: small, medium, and large to treat aortic valve annuli between 21–23 mm, 23–25 mm, 25–27 mm, respectively. Both transapical (28 F) and the transfemoral (18 F) accesses are available for the device implantation. The company recently has developed a 14-Fr expandable introducer sheath, the iSleeve™ that accommodates all three valve sizes. ACURATE Neo2 has shown excellent procedural success, with low mortality rates in an intermediate-risk population at both short and intermediate-term follow-up, and notably low rates of PPI and PVL [26,27]. However, in cases with severe valve calcification, pre- and post-dilatation are highly needed. This is a consequence of the lower radial force of the nitinol frame of the device [28]. However, this characteristic of the device lead to less mechanical trauma to the conduction system and this can be a possible explanation of the lower pacemaker rate of the device [29,30]. Longer follow-up data are needed to confirm the durability of this device. In addition, the range of ACURATE Neo valve sizes does not cover the entire spectrum of annular sizes covered by some of the other current THVs. Finally, the lack of repositionability is a limitation of this device when compared with other supra-annular THVs.

### 2.4. Portico and Navitor SEV

**Figure 4:**
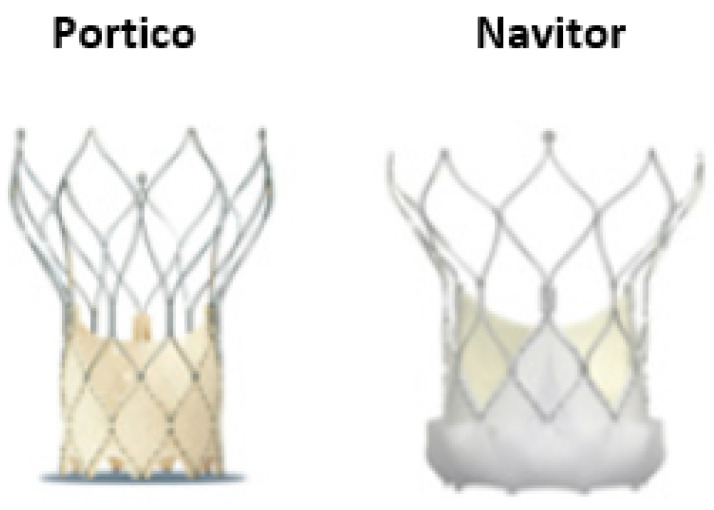
The self-expandable intra-annular Portico and Navitor valve is the only self-expanding valve with intra-annular leaflets and a non-tapered stent which provides uncompromised coronary access.

The re-sheathable and repositionable Portico™ (Abbott Laboratories), is a self-expandable valve with an intra-annular design, providing early valve function and hemodynamic stability throughout the procedure (Figure 4) [31]. The Navitor, a third-generation device, is the successor of the Portico valve. It has intra-annular leaflets and large frame cells, which enhances coronary access in case of future interventions and is currently available in four sizes: 23 mm, 25 mm, 27 mm, and 29 mm. An important advancement of the new valve is the induction of the FlexNav delivery system, a 14F low-profile delivery system with improved deliverability, suitable for cases with small peripheral access (up to 5 mm). A cuffed design with dedicated outer and inner fabric cuffs aims to reduce paravalvular leak and improve sealing and increase deliverability. Furthermore, the intra-annular design preserves future coronary access. Preliminary data showed that residual aortic regurgitation and PVL appear uncommon with Navitor. However, preliminary data highlighted the potential risk of increased rates of permanent pacemaker implantation (PPI) [32]. More data are needed to confirm the short term and long-term outcomes of this device.

### 2.5. Myval BEV

**Figure 5:**
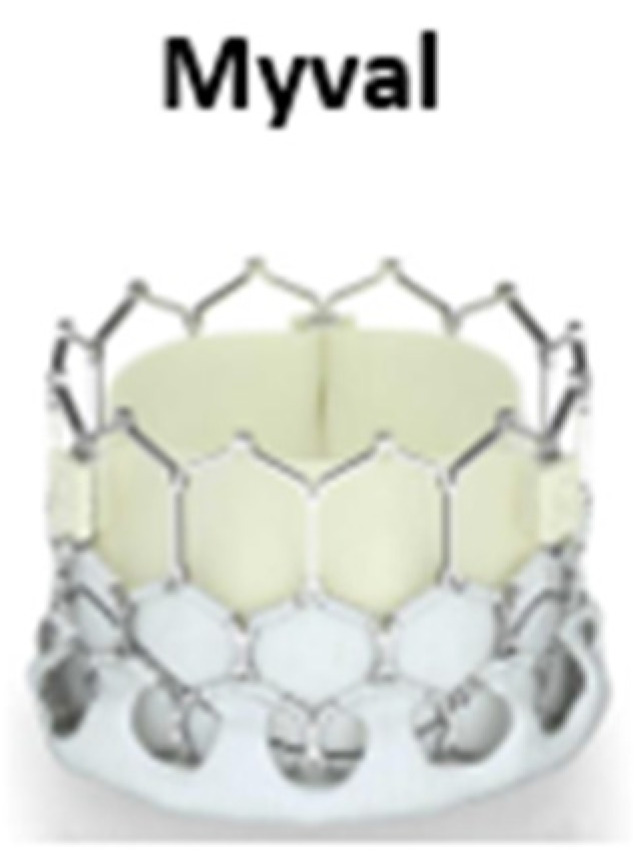
The Myval balloon-expandable valve is made by a nickel-cobalt alloy frame which is created of a single design element open cell hexagons while the remainder has closed cells with higher annular radial force towards the ventricular end.

The Myval THV is a BEV THV. The device is made by a nickel-cobalt alloy frame which is created of a single design element—hexagons (Figure 5). These are positioned in a hybrid honeycomb fashion allowing 53% of the frame to have large open cells towards the aortic end, while the remainder has closed cells with higher annular radial force towards the ventricular end [33]. This design geometry creates a unique dark-light band-like pattern which allows precise positioning, placement, and deployment of the THV across the native annulus. The valve construction material is decellularized bovine pericardium tissue, exposed to an anti-calcification treatment and crafted into a trileaflet valve. As the other BEVs, the lower segment of the valve is covered throughout with a protective sealing cuff of PET in order to reduce the risk of PVL. The Myval THV is manufactured in diameters of 20 mm, 21.5 mm, 23 mm, 24.5 mm, 26 mm, 27.5 mm, 29 mm, and 32 mm. Recent data showed the primary safety and efficacy of the Myval THV with no new pacemaker requirement up to 12-month follow-up [34]. More data are needed to confirm the short-term and long-term results of this new THV.

## 3. Patient-Specific Characteristics

Understanding the advantages and disadvantages of each valve and personalizing the choice of the valve selection based on specific patient clinical and anatomical characteristics is paramount. The most important factors contributing to the valve selection is the size of the aortic annular and the information related to the access route (Table 1). However, an experienced team should not only rely on these features, but also on a holistic impression. Below we describe the most common anatomic and clinical challenges that can contribute to the process of the appropriate valve selection (Table 2).

### 3.1. Patients with a Severely Calcified Annulus

Aortic valve calcification is important for the safe anchoring of the prosthesis onto the aortic annulus. However, excessive aortic valve calcification is related to procedural complications. The most common procedural complications during TAVI are PVL and conduction disturbances. These complications are mainly related to the degree of annular calcification. More infrequent complications, such as aortic annulus rupture and coronary ostia occlusion, are again connected to the burden of aortic valve calcification [35,36].

Implantation of a THV in a very calcified aortic valve may prohibit the full and symmetric prosthesis expansion, contributing to post-procedural PVL. The latter drives for additional post-dilatation, which might increase the risk for life-threatening complications such as aortic annulus rupture, coronary ostia occlusion and stroke [37]. A recent registry compared the incidence of PVL among patients who underwent TAVI with first-generation and new-generation BEVs and SEVs and found lower rates of residual regurgitation in the new generation group (8.3% vs. 22.9%) [38]. However, the use of a newer valve itself does not pre-exist the absence of the PVL or other complications related to the calcified annulus.

The most dramatic complication related to extensive annulus calcification is aortic annulus rupture. This is a life-threatening complication that comprises contained or uncontained aortic annulus rupture, periaortic hematoma, and rupture of the ventricular septum, resulting from disruption of the aorto-ventricular junction after balloon valvuloplasty, valve implantation, or valve post-dilatation because of residual paravalvular regurgitation [39]. Rupture of the aortic annulus remains a major concern, particularly with BEVs. Especially, moderate to severe sub-annular calcification seems to be a strong predictor of annular rupture, particularly when combined with valve or balloon oversizing of more than 20% by area [40,41].

On aggregate, in a scenario of important annulus calcification, deemed to be at high risk of annulus rupture (e.g., a patient with a small, highly calcified annulus), a SEV rather than a BEV may be chosen to reduce the risk of annular rupture (as one of several potential strategies to reduce the risk of rupture). Annular rupture has been observed almost exclusively after the use of a BEV and very rarely after the use of a SEV. However, a SEV design requires a device with high radial strength, to avoid poor anchoring of the device and other potential complications such as prosthesis migration [3]. The latter, according to the TRAVEL registry seems to be more often with the use of SEVs or first-generation TVHs [42]. In this direction, the use of the Evolute R/PRO, a SEV with increased radial force appears as a reasonable option for cases with significant annulus calcification.

### 3.2. Patients with Horizontal Aorta

Aortic angulation (AA) is the angle between the horizontal plane and the aortic annulus plane in a coronal projection [41]. The degree of this angulation can affect the precise positioning of the THV during TAVI making the procedure more challenging, particularly in an extremely angulated or horizontal aorta (HA).

Abramowitz et al. found that increased AA can influence procedural outcomes. An AA ≥ 48° was reported as the threshold to adversely affect the precise deployment of the THV, procedural success, fluoroscopy time, and PVL in patients undergoing TAVI with early-generation SEV THVs but not with BEVs. The advantage of the BEVs in this scenario appears to be related to the shorter stent frame of the BEV, which causes less interaction with the aorta, and to the flexible delivery system and the central marker at the device that facilitates the operator to optimize the coaxial alignment of the prosthesis [42]. Various methods have been developed to facilitate the delivery of a SEV in hostile aorta anatomies and assist the procedure itself. Among them, the use of a snare-assisted valve method [43] or the use of the apical-to-femoral rail technique [44] has been developed. However, both techniques are considered challenging and require an advanced level of expertise.

Gorla et al. studied the impact of AA in SAS patients treated with the second-generation SEVs Portico, Evolut-R, and ACURATE Neo. They demonstrated that in patients with horizontal anatomy (AA > 57°), the rates of moderate/severe PVL were higher in the Evolut-R group (20.8%), whereas device success was comparable among the three devices. AA was a significant predictor of moderate/severe PVLs only in the Evolut-R population. On univariate analysis implantation depth was confirmed among the most significant predictors of moderate/severe PVL [45]. Similarly, the HORSE registry showed that TAVI in patients with an AA more than 49° was associated with worst outcomes. However, among these cases, patients treated with ACURATE Neo valve showed more favourable outcomes in comparison with these cases treated with the EVOLUTE R/PRO THV [46].

Veulemans et al. showed that patients with HA (defined as AA > 51°) treated with new generation SEVs Evolut R and Evolut PRO had an increased rate of stroke (7.1 vs. 2.7%; *p* = 0.033) and 30-day mortality (3.3 vs. 0.4%; *p* = 0.038). The presence of significant calcification of the noncoronary cusp and left ventricular outflow tract (LVOT), the significance of the aortic angulation as well as the need for repositioning manoeuvres were independent predictors for overall adverse outcomes [47]. However, more recent data showed that AA may no longer play a role with the third-generation SEV CoreValve Evolut PRO/PRO+ (Medtronic) THVs. Recently, Medranda et al. showed that extreme AA (defined as AA ≥ 48°) did not affect the success of the procedure, the number of valves used, the severity of the PVL needed for permanent pacemaker implantation, or the rate of in-hospital stroke (or in-hospital death) [48]. The major factors contributing to these good outcomes was the use of the new generation Evolut PRO device that enhances radial force during implantation [49]. However, these are results from a single, high-volume TAVR centre, and results in less experienced centres may differ.

Based on these data, BEV valves with centre markers are considered the first option in patients with increased AA. However, an ACURATE Neo2 or the third-generation Evolut PRO+ device may also be considered safe if a SEV should be selected.

### 3.3. Patients with Extreme Annulus Dimensions or Difficult Sizing

The proper selection of THV according to the dimension of the aortic annulus is fundamental to achieving procedural success and optimal hemodynamic and clinical outcomes after TAVI [50]. Patients with small aortic annulus are at higher risk for patient prosthesis mismatch (PPM). It is well described in large surgical registries and meta-analysis that PPM post-SAVR associated with decreased long-term survival [51,52,53]. Interestingly, this effect of PPM on survival has not been found in early TAVI meta-analysis, but these results require careful interpretation due to shorter clinical follow-up [54]. However, more recent data have shown that PPM post-TAVI leads to higher transvalvular gradients, elevated mortality and heart failure rehospitalization [55] and worst long-term clinical outcomes, especially in patients with low flow gradients [56]. There are also recent reports that pointed out a correlation between PPM with subclinical valve thrombosis [57]. PPM occurs when the EOA of the prosthesis is too small according to the patient’s body size [54,58]. SEV with the supra-annular valve position allows for a larger EOA, lower transvalvular gradients and, consequently less PPM, as compared with BEV, which is characterized by intra-annular position and thereby reduced EOA [59,60,61].

A recent propensity scoring match analysis showed that SEVs are associated with a significantly lower incidence of PPM as compared with BEV in TAVI patients with relatively larger annuli. The difference was mainly driven by patients with larger body sizes (BSA > 1.83 m^2^) [62]. Similarly, Mauri et al. demonstrated a lower incidence of PPM in patients treated with the supra-annular SEV ACURATE Neo, as compared with SAPIEN 3 in patients with a small annulus area (<400 mm^2^) [63]. Likewise, in the CHOICE-Extend registry, TAVI with the second generation SEV Evolute R in small annuli was associated with a lower rate of PPM than with the S3, with no increased risk for PVL [64].

On aggregate, it might be necessary to select the valve type in view of the risk of patient prosthesis mismatch, not only in patients with small annuli, but also in those with relatively large body sizes even if the annulus size is not small. These patients may benefit from TAVI with a SEV device while keeping in mind a higher possible risk of permanent pacemaker implantation, and post-procedural PVL.

On the opposite end of the spectrum, individuals with larger annulus (usually defined as an area >680–700 mm^2^), are usually lacking multiple THV options. For these patients with annulus dimensions beyond the optional range, a recent multicentre observational study suggested that TAVI with a 29-mm SAPIEN 3 THV using overexpansion (by adding volume—1 to 5 mL extra—during initial deployment, with nominal filling in the remaining 43%) was safe and effective up to 1 year with acceptable rates of PVL and new permanent pacemaker implantation [65,66]. Another option for this scenario, is the use of the newer-generation balloon-expandable valve Myval. Myval BEV offers two additional sizes (30.5 and 32.5 mm), covering larger areas up to 840 mm^2^ [34]. Finally, the 34-mm Evolut R XL is at present another choice in patients with extremely large valves, as it can be used for annuli with diameter of up to 30 mm and perimeter up to 94.2 mm [67].

Finally, we cannot ignore that apart from cases with extreme (very small or very large) annulus dimensions, operators may face challenges in cases of difficult measuring and sizing of respective annuli. Usually, these are cases with specific anatomical characteristics of the aortic root including cases with tremendous annulus calcification, bicuspid aortic valve anatomy, increase calcification of the LVOT and an eccentric aortic annulus. In such cases, suboptimal THV sizing can be associated with lower rates of device success. Data from the Bern registry showed that among the patients with difficult valve sizing, oversizing was linked with higher rates of device success compared with undersizing [68]. In oversizing scenarios, SEV technologies are preferred over BEV as in these cases there is always an elevated risk of annulus rupture, and the hazard of aortic annulus rupture is related to the degree of oversize of BEV [40]. In cases of undersizing, however, novel THV technologies using dedicated sealing skirts or cuffs may be more effective in minimising PVL, and improving procedural outcomes [69,70].

### 3.4. Patients with Bicuspid Aortic Valve

Bicuspid aortic valve (BAV) anatomy is the most common congenital cardiac condition, affecting 1–2% of the general population. In addition, BAV (both type 0 and I combined) is frequently associated with concomitant aortic pathology, including stenosis, regurgitation, aortopathy, and aortic dissection [71]. Traditionally, SAVR is the first-choice treatment for cases of BAV with valvular dysfunction requiring treatment. Of note, the degree of aortic root dilatation determines the type of operation. However, the recent expansion of TAVI indications to lower-risk and younger patients will lead to increased use of TAVI for elderly patients with BAV, while BAV is responsible for almost 50% of patients undergoing SAVR or the Ross procedure at younger ages [72].

Traditionally, BAV was considered a contraindication for TAVI, and BAV patients were excluded from all landmarks RCTs. TAVI for BAV stenosis presents both anatomic and clinical challenges. The first challenge in BAV cases is the large aortic annulus. The dimensions of all components of the AV are generally larger in BAV than in tricuspid AV (TAV), increasing the likelihood of having an annulus size outside of the range covered by currently available THV [73]. The BAV has a similar circular geometry of the aortic annulus as that of the TAV. However, the supra-annular geometry, especially at Valsalva’s sinus level, is often more elliptical [74]. This can create a second challenge in BAV patients, as the suboptimal expansion of the prosthesis in an elliptical orifice with two commissures, can result in significant paravalvular regurgitation. Assessing the stent frame expansion in two orthogonal views can unmask areas of under-expansion and may urge post-dilatation [75].

Furthermore, the complex BAV aortic shape creates the narrowest dimension and point of highest resistance of the AV above the annulus at the commissural level, which may result in a less circular deployment. This problem occurs as another concern in the procedure, e.g., possible oversizing of the THV can result in annulus injury if sized according to the annulus dimensions [76]. Finally, BAVs are often heavily calcified, accompanied by raphe (fusion between adjacent cusps in type I BAV), and have concomitant aortopathy (dilatation of the ascending aorta), which may require additional surgical treatment of the aorta [77].

Data from large registries comparing patients with BAV vs. TAV AS showed no significant difference in 30-day or 1-year mortality but presented an increased 30-day risk for stroke for patients treated with the third-generation BEVs [78]. Yoon et al. showed that in patients treated with early-generation devices, the cases of BAV AS had more frequent complications including aortic root injury with BEV, and moderate-to-severe PVL with SEV. However, overall procedural results were comparable across different prostheses among patients with new-generation devices. Compared with TAV AS, TAVI in BAV AS was associated with a similar prognosis (2-year all-cause mortality rate 17.2% vs. 19.4%, respectively, *p* = 0.28), and similar success rate with new generation devices (95.1% vs. 97.8% in tricuspid and bicuspid aortic valves, respectively, *p* = 0.13) compared to previous generation prosthesis (78.4% vs. 86.9% in TAV and BAV aortic valves, respectively, *p* < 0.01) [79].

Similar results resulted from the BEAT registry, which proven the feasibility of both Evolut R/PRO and SAPIEN 3 valves in BAV anatomy (Evolut R/PRO = 84.4% versus SAPIEN 3 = 85.7%, *p* = 0.821). Similarly with previous evidence, an elevated risk of moderate-severe PVL was detected in the Evolut R/PRO group at 12-months follow-up in the matched cohort (Evolut R/PRO 10.5% versus SAPIEN 3 4.2%, *p* = 0.077) [80].

More recently, Forrest et al. showed that transcatheter aortic valve replacement with new generation SEV Evolut or Evolut PRO in low-surgical risk patients with BAV stenosis succeeded encouraging 30-day results, with a device success up to 95.3% (95% CI, 90.5–98.1%), and low rates of stroke and death at 1.3% (95% CI, 90.5–98.1%). Of note, the rate of permanent pacemaker implantation was 15.1% [81].

Similar results are also coming for series with BAV patients treated with new generation BEV SAPIEN 3. In selected cases of low-surgical-risk with BAV, the use of a SAPIEN 3 valve showed similar results to a matched group of patients with tricuspid valve. According to this study, both the primary endpoint between BAV and TAV (10.9% vs. 10.2%; *p* = 0.80) and the rates of the individual components (death: 0.7% vs. 1.4%; *p* = 0.58; stroke: 2.1% vs. 2.0%; *p* = 0.99; cardiovascular rehospitalization: 9.6% vs. 9.5%; *p* = 0.96) were similar [82].

In conclusion, TAVI in BAV is feasible, and implants are recommended to be rather high, remaining across the annulus. For this purpose, using a third-generation SEV with the option of repositioning seems the ideal option. However, current evidence supports that also the new-generation BEV (SAPIEN 3) may be used in BAV with a lower incidence of PVL and permanent pacemaker implantation (PPI) when compared with SEVs. Of note, only SAPIEN 3 BEVs and Evolut R/PRO SEVs received approval for use in BAVs.

### 3.5. Patients with Limited Transfemoral Access

Transfemoral access (TF) is used in more than 90% of cases in centres where the procedure is conducted regularly [83]. The miniaturization of introducer sheaths is remarkable. With 20–24 Fr introducer sheaths in the first TAVI years, now most of the devices are 14–16 Fr compatible, reducing the rate of vascular complications. The smaller profile of latest-generation devices has increased the percentage of patients eligible for TF access with a minimum required vessel size currently down to 5–5.5 mm. Additionally, the use of intravascular lithotripsy and a great variety of closure devices facilitate TF access further reducing vascular complications [84,85]. However, there are still many patients with hostile TF access due to excessive calcification, atherosclerotic disease, tortuosity, severe kinking, or insufficient vessel calibre. These patients may require an alternative access route, among the various alternatives, including transapical, trans-subclavian/axillary, transcarotid, direct aortic and, more recently, transcaval [86].

Pre-procedural planning is paramount and has a direct impact on the feasibility of TF TAVI. Vascular minimal diameter, tortuosity, and extent and distribution of calcification are major predictors of vascular complications and the success of the procedure [87,88]. The induction of newer low-profile devices with smaller sheath sizes (16 and 14 Fr) has reduced a lot the rates of vascular access-related complications. Furthermore, the use of expandable sheaths (such as the Edwards eSheath^®^ or the Boston iSleeve^®^) has significantly decreased the rate of vascular complications. These sheaths permit a continuous expansion of the system during the advancement of the valve [89]. Especially in unclear anatomies—such as cases with severe calcification in combination with severe kinking—the advancement of “sheathless” TAVI valves, such as the Abbott Navitor FlexNav™ or the Medtronic InLine Sheath might be challenging. In these complex anatomies, the use of an expandable sheath can be a helpful solution to successfully assist transfemoral access. Then, an expandable sheath can be appraised, and the prosthesis can be reinserted. At present, all the new generation commercially available systems, are associated with lower incidence of major vascular complications [89,90,91].

### 3.6. Patients at High Risk for Conduction Abnormalities

Even though the need for a permanent pacemaker has progressively decreased over the years, currently varying between 5–25%, the need for permanent pacing remains a major TAVI disadvantage [92]. The most common risk factors for permanent pacing, include pre-existing rhythm abnormalities, anatomical factors, and technical factors [93]. Pre-procedural planning, focusing on the identification of these risk factors is crucial to minimise the risk or even to stratify for upfront pacemaker insertion.

The principal independent predictors for permanent pacing post-TAVI are age, pre-existing RBBB, LBBB, SEVs, and valve implantation depth [94]. Undoubtedly, the risk for new PPI after TAVI is strongly linked to the valve type. The use of SEVs was consistently recognised in many randomised trials, observational trials and meta-analyses as a major independent predictor [95]. The elevated risk of high degree AV block has been linked to both the design of the valve and the depth of implantation into the LVOT, which may contribute to an elevated risk of injury to the AV node and left bundle branches [96,97,98,99]. The BEVs appear to have a lower permanent pacing rate with a risk for the new-generation Edwards Lifesciences SAPIEN 3 device ranging between 2.4% and 24% [100]. Similarly, the novel Myval BEV is associated with a low rate of early conduction disturbances [101]. This advantage of BEVs over SEVs in post-TAVI PPI, seems to be maintained even with the last-generation SEVs in lower-risk patients [102,103].

For SEVs, the permanent pacing rate is variable, with a higher incidence with the Medtronic CoreValve Evolut R and the new CoreValve Evolut R PRO (between 6.1% and 26.7%) [104], and an even higher permanent pacing risk with the Portico THV (PPI rate of 21.9% and 27.7% in propensity-matched and randomized studies, respectively) [105] and a lower incidence with the Boston Scientific’s 13CCURATE Neo valve (between 2.3% and 10.2%) [105,106]. The SCOPE 2 randomised study compared the clinical outcomes of the new generation SEVs Evolute R and Accurate Neo. The 30-day rates of new permanent pacemaker implantation were 10.5% in the 13CCURATE Neo group and 18.0% in the CoreValve Evolut group (*p* = 0.0027) [107]. The same low risk of permanent pacing with the 13CCURATE Neo and Neo2 has been recognised in observational studies and registries [25,27,108]. This relative lower risk of PPI appears to be related to both lower radial strength of the nitinol frame, and limited extension to the membranous septum that may lead to less mechanical injury to the conduction system [109]. Thus, in individuals who are considered high risk for conduction abnormalities and believed to carry an elevated risk for PPI post-TAVI, the use of a BEV seems a reasonable option. The use of the new generation 13CCURATE Neo 2, a device with lower radial strength, may be considered as a safe option in patients at high risk for conduction abnormalities.

### 3.7. Patients with a Need for Future Coronary Engagement

Coronary artery disease is present in almost 60–70% of TAVI patients [110]. Considering that even younger patients are nowadays treated with TAVI, easy coronary access for future coronary intervention is becoming important. The obvious preference therefore is the use of a BEV. This can be easily understood by the differences in the geometry and the positioning between a BEV and a SEV. A BEV deploying intra-annularly, has the clear advantage of less interaction with both coronary ostia avoiding the problem of future engagement. Early studies described a very fluctuating rate of coronary access difficulties after TAVI ranging from 50% to 100% for both types of the valve [111]. However, a more recent study revealed coronary re-access difficulties are more common with SEV than with BEV. Based on the study from Ochiai et al., the left and right coronary artery ostia were in an unfavourable position in 34.8% and 25.8%, respectively, in these patients where a SEV Evolute R or PRO has been used. In contrast, in the BEV SAPIEN 3 population, the left and right coronary ostia were unfavourable in 15.7% and 8.1% of cases, respectively [112]. Coronary access after a BEV implantation is relatively easy due to the shorter stent frame and sub-coronary position of the valve stent [113].

Coronary cannulation and catheter manipulation may be challenging after TAVI with a SEV, due to the large, stented part of the valve that located across coronary ostia [114]. The recent RE-ACCESS study showed that the combination of Evolut SEV, a higher THV-sinus of Valsalva relation, and implantation depth predicts the risk for unsuccessful coronary cannulation after TAVI with high accuracy [115]. However, implantation of a supra-annular SEV THVs with the method of commissural alignment, can overcome this challenge. Commissural alignment focuses on reduced neo-commissure overlap (less interaction of the valve cells) with the coronary ostia. This method prevents coronary access optimizing procedural outcomes and increasing the options for possible future interventions [116,117]. The ALIGN TAVR and ALIGN-ACCESS studies confirmed that commissural alignment improves the rate of selective coronary access after TAVI with supra-annular THVs [118,119].

In conclusion, valves with lower stent frames and intra-annular leaflets have a lesser possibility of causing coronary obstruction and should be considered for younger patients. If an SEV should be selected, the implantation with the technique of the commissural alignment, preventing valve implantations with overlap between the coronary ostia and valve commissures, is another option.

### 3.8. Life-Time Management of Patients with Severe Aortic Stenosis

Apart from the coronary re-access post-TAVI, the other important potential problem in the group of relatively young patients receiving a TAVI valve is the need for a future valve intervention. Given proper time, the initial THV will start to degenerate and the longer life expectancy of the patients who are currently treated with TAVI could result in an increased rate of future valve re-intervention. TAVI-in-TAVI (or in other words redo-TAVI) is an attractive and feasible method but carries a risk of coronary obstruction [120].

The matter of coronary obstruction and coronary re-access is becoming even more important in these future TAVI-in-TAVI therapies. If a tall frame THV (e.g., a SEV) has been used at the time of the first intervention and the upper part of the initial valve is above the sinotubular junction (STJ), the future TAVI-in-TAVI may be prohibited, because the displaced leaflets of the original THV may possess the coronary sinuses, occluding the coronary ostia [121]. Patients with low coronary ostia and narrow sinus of Valsalva are at a higher risk of coronary obstruction in this scenario [122]. Therefore, TAVI-in-TAVI on supra-annular THVs is potentially dangerous as the new valve tends to push the prior leaflets against the original frame that extends above the STJ, and theoretically can obstruct the coronary blood flow and restrict catheter access [123]. Consequently, in a young patient with a future need for TAVI-in-TAVI intervention, the use of an intra-annular valve should be considered.

Various approaches have been developed in order to achieve new THV commissural alignment and facilitate future coronary access [117,118]. Furthermore, the on-purpose scallop laceration of a bioprosthetic (or even native) valve to prevent coronary artery obstruction during TAVI (the BASILICA method) has been developed as an alternative to limit the risk of iatrogenic coronary obstruction and facilitate the coronary access, in patients undergoing TAVI-in-TAVI [124]. Other peri-procedural methods to minimize the risk of coronary obstruction during TAVI-in-TAVI involve the protection of the coronary with a guidewire and an uninflated balloon or the chimney technique [125]. After all, the ‘a priory’ use of dedicated protocols based on CT scan simulation can predict whether a patient can have another TAVI in his lifetime, and for those who cannot, it may be wise to undergo surgery first [126].

## 4. Conclusions

In conclusion, understanding each THV’s advantages and disadvantages is paramount for the correct valve choice, minimising early adverse outcomes and maximising long-term clinical success. Many centres acquire experience using only one type of valve to achieve high procedural success even though at least having experience with a BEV and a SEV concept is desirable. However, tailoring the selection of a TAVI device to suit the patient’s individual clinical and anatomical characteristics may be essential for optimized outcomes. The selection of a THV should not only rely on the range of valve sizes available, the dimensions of the delivery device versus the native vessels, and the possible access routes, but also on specific clinical scenarios, such as those described above. Thus, in the end, one valve does not suit all cases.

## 5. Future Directions

There are currently no clear guidelines regarding the choice between SEVs and BEVs. Future studies directed towards a head-to-head comparison of commercially available THVs in specific scenarios, are more than welcome and needed. An example is the upcoming randomized SMall Annuli Randomized to the Evolut or SAPIEN (SMART) trial (NCT04722250) that aims to evaluate the TAVI with SEV and BEV in subjects with a small aortic annulus and symptomatic severe native aortic stenosis. Such evidence would be substantial in the most suitable selection of the TVHs.

## Figures and Tables

**Table 1:** Synopsis of the commercially available Transcatheter Heart Valves (THVs) with FDA and/or CE Mark approval.

THV	Frame Material	Leaflet Material	Supraannular or Intraannular	Repositionability	Retrievability	Available Valve Sizes Based on the Correspondence Aortic Valve Area (in mm^2^) for BEVs or Aortic Perimeter (in mm) for SEVs	Available Sheath Sizes	Delivery Routes	Approval
					BEVs				
Sapien 3	Cobalt-chromium	Bovine pericardium	Intraannular	No	No	20 (273–345 mm^2^), 23 (338–430 mm^2^), 26 (430–546 mm^2^), 29 (540–680 mm^2^)	14 F (20, 23, 26 mm), 16F (29 mm)	TF, TA, TAo	FDA, CE Mark
Sapien 3 Ultra	Cobalt-chromium	Bovine pericardium	Intraannular	No	No	20 (273–345 mm^2^), 23 (338–430 mm^2^), 26 (430–546 mm^2^), 29 (540–680 mm^2^)	14 F	TF, TA	FDA, CE Mark
Myval	Nickel-cobalt	Bovine pericardium	Intraannular	No	No	20 (270–330 mm^2^), 21.5 (314–380 mm^2^), 23 (360–440 mm^2^), 24.5 (410–500 mm^2^), 26 (460–560 mm^2^), 27.5 (510–630 mm^2^), 29 (570–700 mm^2^) 30.5 (630–770 mm^2^), 32 (700–840 mm^2^)	14 F	TF	CE Mark
					SEVs				
Evolut PRO	Nitinol	Porcine pericardium	Supraannular	Yes	Yes	23 (56.5–62.8 mm), 26 (62.8–72.3 mm), 29 (72.3–81.7 mm), 34 (81.7–94.2 mm)	16 F	TF, TAo, SC	FDA, CE Mark
Evolut PRO +	Nitinol	Porcine pericardium	Supraannular	Yes	Yes	23 26, 29, 34	14 F (23, 26, 29 mm), 16F (34 mm)	TF, TAo, SC	FDA, CE Mark
ACURATE neo2	Nitinol	Porcine pericardium	Supraannular	No	No	Small (66–72 mm), Medium (72–79 mm), Large (79–85 mm)	14 F	TF, TA	CE Mark
Portico	Nitinol	Bovine pericardium	Intraannular	Yes	Yes	23 (60–66 mm), 25 (66–73 mm), 27 (72–79 mm), 29 (79–85 mm)	18 F (23, 25 mm), 19 F (27, 29 mm)	TF, TAo, TAx, SC	CE Mark
Navitor	Nitinol	Bovine pericardium	Intraannular	Yes	Yes	23 (60–66 mm), 25 (66–73 mm), 27 (72–79 mm), 29 (79–85 mm)	14 F (23, 25 mm), 15 F (27, 29 mm)	TF, TAo, TAx, SC	CE Mark

TF—Transfemoral, TA—Transapical, TAo—Transaortic, TAx—Transaxillary, SC—Subclavian, CA—Carotid. FDA Approval—approved for use by the United States Food and Drug Administration. CE Mark Approval–approved for use across all EU member states and European Economic Area by the European Commission.

**Table 2:** Proper device selection based on specific patient anatomic and clinical characteristics.

Scenario	Risk	Recommended	Valve
		BEV	SEV
*Anatomic Characteristics*			
Severely calcified annulus	Elevated risk for annulus rupture, PVL, or stroke		√
Horizontal Aorta	Difficult delivery and precise positioning of the valve	√	
Small annulus size	Elevated risk for PPM and annulus rupture		√
Large annulus size	Limited commercially available options	√	
Bicuspid Aortic Valve	Difficult sizing and higher risk of annulus rupture and PVL	√	√
Limited transfemoral access	Elevated risk for vascular complications		√
*Clinical characteristics*			
Pre-existing risk factors for conduction abnormalities	High risk for post-procedural permanent pacing	√	
Concomitant Coronary Artery Disease	Future need for coronary engagement	√	
Grater Life-expectancy	Possible need for aortic valve re-intervention (TAVI-in-TAVI)	√	
Patients with reduced ejection fraction of the left ventricle	Poor tolerance to rapid pacing		√

BEV—Balloon expandable valve, SEV—Self expandable valve, PVL—paravalvular leak, PPM—patient prosthesis mismatch, TAVI—transcatheter aortic valve implantation.
